# Functional and Radiological Outcomes of Distal Femur Fractures Treated With a Combination of Supracondylar Nails and Lateral Condylar Locking Plates

**DOI:** 10.7759/cureus.105075

**Published:** 2026-03-11

**Authors:** Nilesh Joshi, Darshan Sharma, Shantanu Deshkmukh, Sushil Mankar, Pallav P Agrawal, Kaustubh Zodey, Adilagna Das, Prithveesh Narang, Pranav Datta

**Affiliations:** 1 Orthopaedics and Traumatology, N. K. P. Salve Institute of Medical Sciences and Research Centre and Lata Mangeshkar Hospital, Nagpur, IND; 2 Orthopaedic Surgery, N. K. P. Salve Institute of Medical Sciences and Research Centre and Lata Mangeshkar Hospital, Nagpur, IND; 3 Orthopaedics, N. K. P. Salve Institute of Medical Sciences and Research Centre and Lata Mangeshkar Hospital, Nagpur, IND; 4 Medicine and Surgery, Jawaharlal Nehru Medical College, Alwar, IND

**Keywords:** distal femur fracture, intramedullary nailing (imn), locking plate fixation, nail-plate construct, patient-reported outcomes (pros)

## Abstract

Introduction

Distal femur fractures are associated with significant morbidity, particularly in elderly patients, and achieving stable fixation that permits early mobilization remains challenging. The nail-plate construct (NPC) combines the biomechanical advantages of intramedullary nailing and lateral locking plate fixation and may provide improved stability and functional outcomes.

Methods

This prospective observational study evaluated adult patients with acute distal femur fractures treated using an NPC at a tertiary care center. Thirty-two patients were included and subgrouped based on age (<65 years and ≥65 years). Radiological union and malunion at one-year follow-up were the primary outcomes. Secondary outcomes included postoperative mobilization, length of hospital stay, complications, and patient-reported outcomes measured using the EuroQol five-dimension five-level (EQ-5D-5L) questionnaire.

Results

The median age of the cohort was 70.5 years (range: 30-91), with the majority aged ≥65 years. Low-energy mechanisms accounted for 28 (87.5%) of fractures, and 30 (93.8%) were closed injuries. At one-year follow-up, radiographic union was achieved in all patients, with no cases of malunion or implant failure. Early postoperative mobilisation was feasible across age groups. The mean EQ-5D-5L index value demonstrated favourable functional recovery, with most patients reporting no or only slight problems with mobility, self-care, and usual activities. Postoperative complications occurred predominantly in the elderly subgroup, with a small proportion requiring reoperation.

Conclusion

NPC fixation for acute distal femur fractures resulted in excellent radiological union, early mobilization, and favorable patient-reported outcomes at one year. This technique appears to provide stable fixation that supports early rehabilitation, particularly in elderly patients. Larger comparative studies with longer follow-up are required to further define its role relative to isolated fixation methods.

## Introduction

Distal femur fractures are caused by high-energy injury, having high morbidity and mortality rates [1]. The age distribution is usually bimodal in nature. In young patients, it is due to high-energy trauma like road traffic accidents, assaults, etc., and in the older age group, it is due to osteoporosis-related fractures, including trivial trauma [2]. The one-year mortality rate for distal femur fractures in elderly patients was 13.4% [3]. The prevalence of symptomatic venous thromboembolism in distal femur fractures is high at 15.3% [4]. Improved postoperative range of motion is seen in surgically managed distal femur fractures [5]. Postoperative complications, hospital stay, and mortality rates are reduced due to early mobilisation and weight-bearing ability [6]. Locked lateral plating (LP) and intramedullary nailing (IMN) are widely accepted surgical options for the treatment of distal femoral fractures [7]. However, limitations have been reported when these techniques are used in isolation. In elderly patients, difficulty in complying with partial or non-weight-bearing protocols may lead to extended hospitalization, increased healthcare costs, and a higher risk of hospital-acquired complications [8].

When lateral LP or IMN are used as standalone fixation methods, reported nonunion rates range from 2.8% to 21% [8,9]. Treatment of distal femur fractures with isolated LP fixation has been associated with malunion rates of approximately 7.6% and secondary surgical intervention rates of 13.3%. Similarly, IMN has demonstrated higher malunion rates of up to 16.4%, with reoperation rates reported at 9.1% [10]. Dual plate fixation has been proposed as an alternative strategy; however, studies have reported nonunion and delayed union rates of 12.5% and 33.3%, respectively [11].

Recently, a nail-plate construct (NPC) has been described for distal femur fracture, which combines both LP and IMN, which are more advantageous for fixation. Various benefits of this fixation are that it reduces the risk of non-union and malunion and also gives early range of motion in patients without constraining weight bearing in patients. As it gives better postoperative mobility, various postoperative morbidity and mortality can be reduced.

The objectives of this study were to: (1) evaluate radiological union and malunion rates in distal femur fractures treated with a combined NPC; (2) assess patient-reported outcome measures (PROMs) and postoperative mobility at one-year follow-up; and (3) document morbidity and mortality outcomes in this patient population.

## Materials and methods

This prospective observational study was conducted at a tertiary care hospital in Maharashtra, India, between June 2022 and June 2024. Patients were eligible for inclusion if they were aged >18 years, had sustained acute distal femur fractures classified as AO/Orthopaedic Trauma Association (OTA) 33-A, 33-B, or 33-C, and were treated using the NPC technique. Patients with periprosthetic distal femur fractures or those undergoing revision surgery for malunion or nonunion were excluded. Exclusion of malunion and nonunion cases was intended to specifically evaluate the outcomes of NPC fixation in the acute fracture setting. Previous studies have reported a 100% union rate with the use of NPC fixation in revision surgery for distal femur nonunion [12].

**Figure 1:**
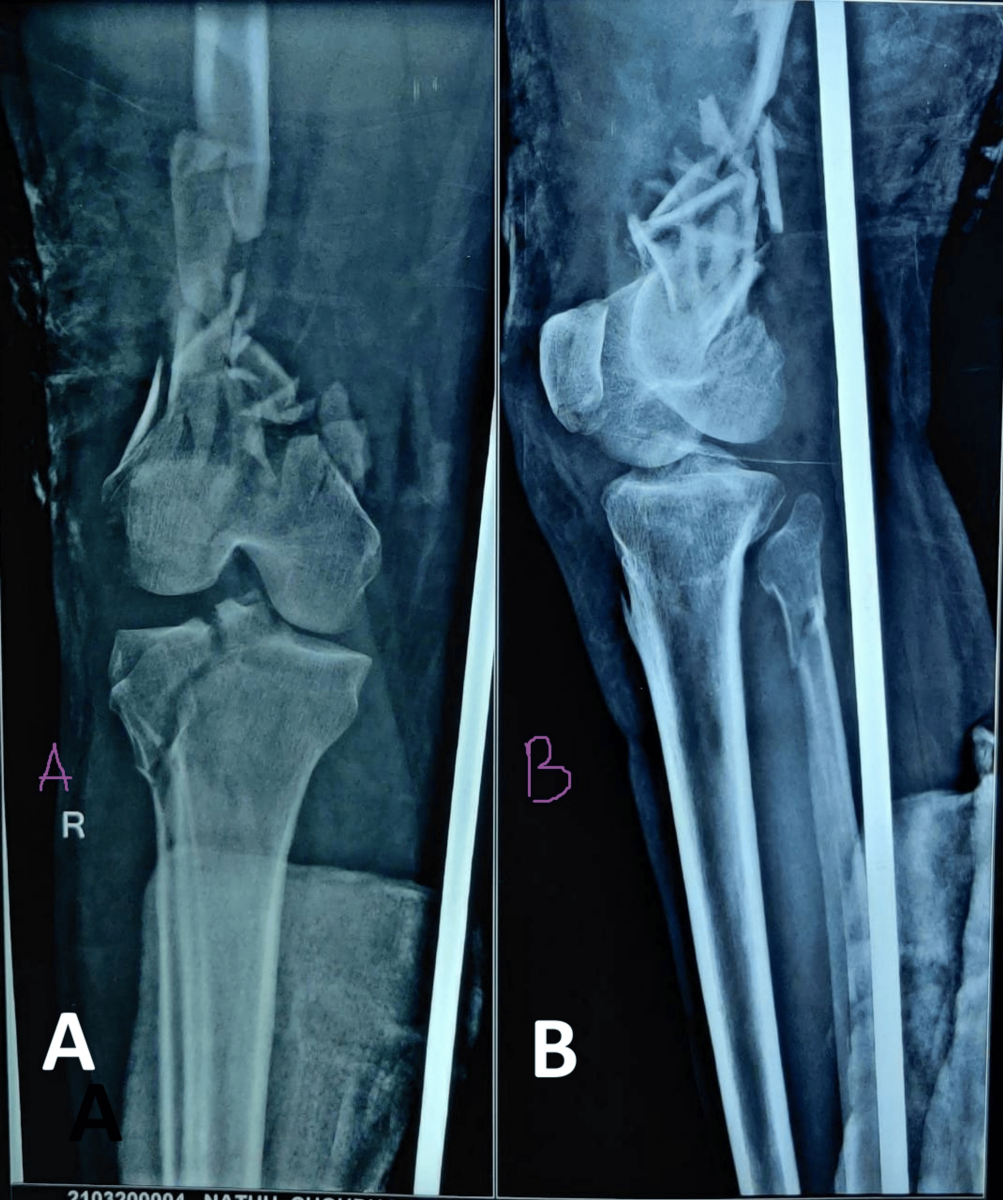
Preoperative radiograph of distal femur fracture (AO/OTA33-C2-3). A: anteroposterior view; B: lateral view

Patient demographic information, like age, sex, and body mass index, was noted. Preoperative radiographs were taken (Figure 1). The functional status of the patient before injury was recorded, including assisted walking or requirement for any aids. Mode of injury, pathological or non-pathological fracture, and any requirement for bone graft were recorded.

Ethics approval was obtained from the Institutional Ethics Committee of N. K. P. Salve Institute of Medical Sciences and Research Centre and Lata Mangeshkar Hospital (approval LMH/IEC/1/2022). This was a prospective observational, non-interventional study involving standard-of-care treatment only. No additional procedures or interventions were performed for research purposes. Informed verbal consent was obtained from all participants for prospective data collection, radiographic evaluation, and assessment of functional outcomes at one-year follow-up

Surgical technique

The NPCs were secured to each other by inserting at least one locking screw through the corresponding distal locking holes of both implants. This combination will give a more stable and stronger construct than using these implants alone. Depending on the fracture configuration, nailing can be done in either an antegrade or retrograde fashion.

Patient position and approach

All patients were positioned in the supine position on a standard radiolucent operating bolster, which was kept beneath the knee. Incision was taken on the lateral parapatellar for intra-articular distal femur fracture for reduction and plate fixation.

For retrograde femoral nailing, a midline incision with medial parapatellar approach was used, and for anterograde femoral nailing standard lateral approach with trochanteric entry was done.

Fixation technique

Fracture reduction was achieved using either direct or indirect techniques through a parapatellar or lateral approach, followed by temporary fixation. Adequacy of reduction was confirmed fluoroscopically. The NPC was assembled by inserting a locking screw through the distal locking holes of both implants. Using either a targeting jig or the fluoroscopic “perfect circle” technique, a temporary K-wire was placed through the distal locking hole of the intramedullary nail. The K-wire was left protruding laterally and used as a guide for positioning the lateral locking plate through the corresponding locking screw hole, thereby facilitating accurate alignment of the distal locking holes of the nail and plate for insertion of the combined distal locking screw. Only a few authors have described this technique. This technique has been described by surgeons as user-friendly and reduces the requirement of intraoperative C-arm radiographs. However, in this study, examination of fluoroscopy use and operative time was not considered. In this study, an intra-articular fracture of the distal femur was treated with NPC with a one-year follow-up radiograph with union.

Postoperative protocol

**Figure 2:**
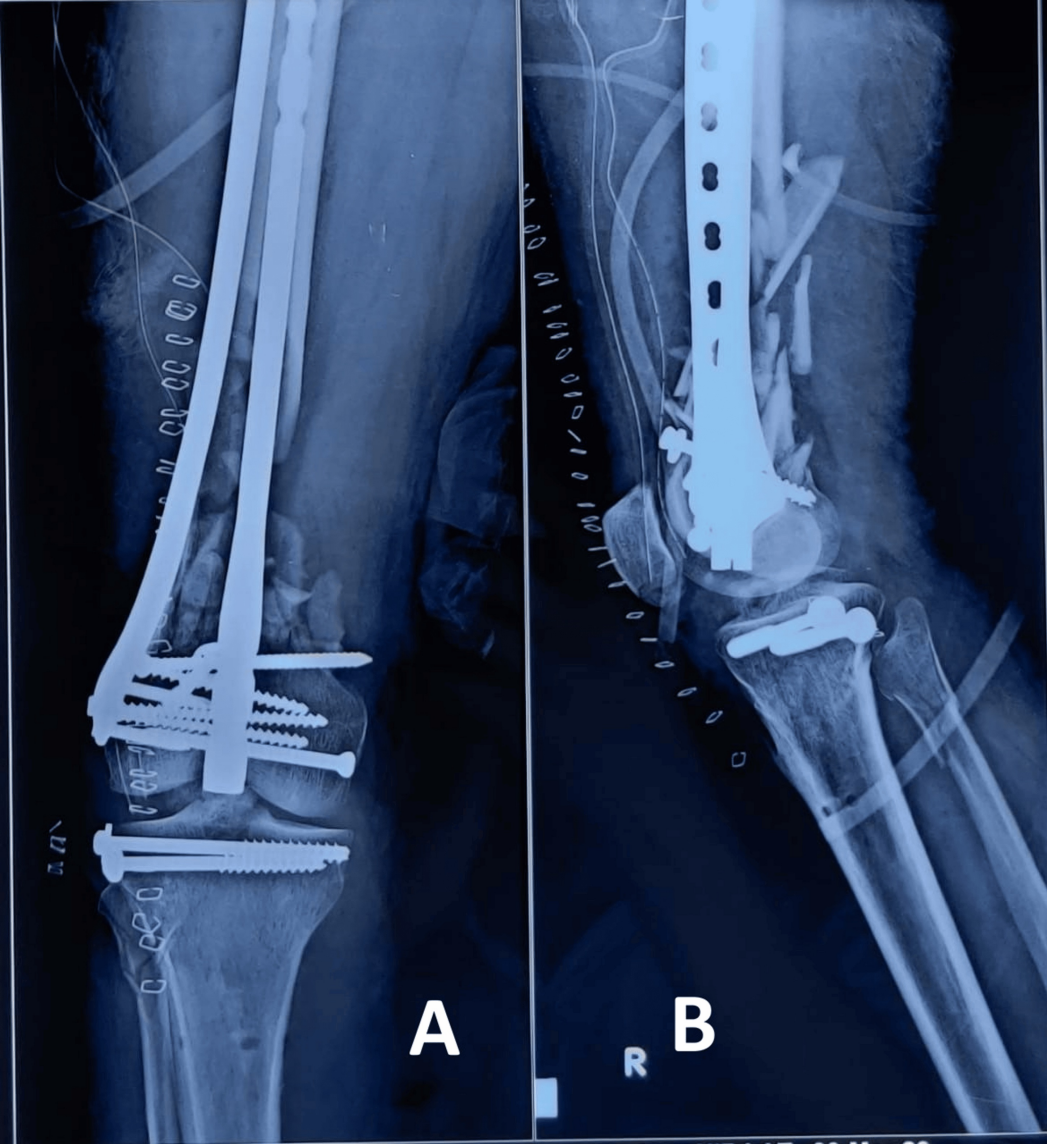
Immediate postoperative X-ray of distal femur fracture operated with nail-plate construct (NPC) fixation. A: anteroposterior view; B: lateral view

All patients were given deep vein thrombosis (DVT) prophylaxis perioperatively, and on induction, they were given prophylactic antibiotics. Postoperatively, X-rays were taken (Figure 2), and all patients were motivated for hip and knee exercises as tolerated by the patient.

Outcome measures

**Figure 3:**
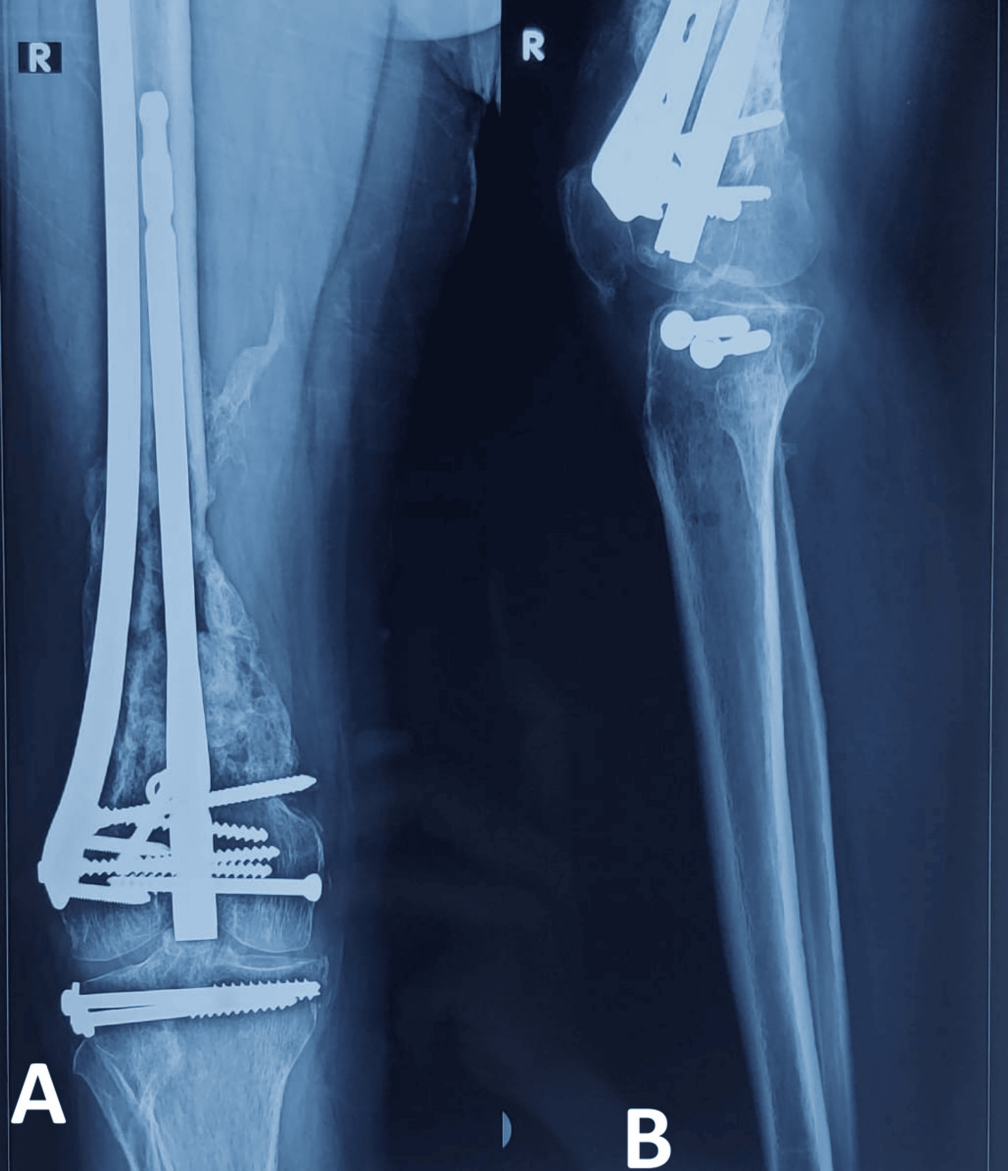
One-year follow-up of distal femur nail-plate construct (NPC) fixation. A: anteroposterior view; B: lateral view

At one-year follow-up, the outcome is mainly measured by radiographic union and malunion (Figure 3). One-year follow-up was selected as it was the minimum follow-up taken in previously related studies on NPC. Modified Radiographic Union Scale in Tibial (RUST) fracture was used to assess radiographic union done by an experienced orthopaedic surgeon. A modified RUST score ≥11 suggests radiological union in the distal femur fracture [13]. Malunion can be defined compared to the previous study as ≥5 [10].

PROMs were assessed using the EuroQol five-dimension five-level (EQ-5D-5L) questionnaire, a validated instrument for evaluating health-related quality of life across five domains: mobility, self-care, usual activities, pain/discomfort, and anxiety/depression [14]. The EQ-5D-5L is a product of the EuroQol Group and was used with the permission of the EuroQol Research Foundation.

Postoperative mobility compared to preoperative mobility, mobilisation done first time after operation, hospital stay duration, postoperative weight bearing status, and any postoperative complications were secondary outcomes. 

Statistical method

Data were reported with mean and standard deviation (SD). No inferential statistical comparisons between age subgroups (<65 years vs ≥65 years) were performed. Given the relatively small sample size and unequal subgroup distribution, the study was not adequately powered to support reliable hypothesis testing. Therefore, subgroup findings are presented descriptively to avoid potential overinterpretation. Radiographs were considered showing “union” with RUST score ≥11 and as ”nonunion” when score <11 [13]. Malunion was considered when the union was ≥5^o^ from anatomical alignment [10]. These data analyses were done using Microsoft Excel (2021; Redmond, WA, USA).

## Results

Thirty-four patients were initially identified for the study; two were excluded - one due to revision surgery for infected distal femur nonunion and the other due to loss to follow-up before one year. Among 32 patients, subgroups were made depending on patient age, as ≥65 years and <65 years of age.

Demographics and follow-up

Patient-related demographics are given in Table 1. Of the 32 patients, 24 (75%) were ≥65 years of age. Patients of the younger age group had a history of road traffic accidents, while older ones had trivial trauma like falling from bed. AO/OTA classification-wise distribution are shown in Table 2.

**Table 1:** Patient Demographics

Baseline Variable	All Patients (n = 32)	Age <65 Years (n = 8)	Age ≥65 Years (n = 24)
Age (years), median (range)	70.5 (30–91)	42.8 (30–56)	74.5 (68–91)
Female sex, n (%)	1 (3.1)	0 (0)	1 (4.2)
Body mass index (kg/m²), mean (SD)	28.1 (5.13)	27.1 (4.69)	29.3 (5.93)
Low-energy mechanism, n (%)	28 (87.5)	4 (50.0)	24 (100)
Closed fracture, n (%)	30 (93.8)	6 (75.0)	24 (100)

**Table 2:** AO/OTA Classifications of Fractures

AO/OTA classification	All ages	Age < 65years	Age ≥ 65
33-A1	6	2	4
33-A2	2	2	0
33-A3	2	2	0
33-C1	4	0	4
33-C2	10	0	10
33-C3	8	2	6

The mean follow-up period of patients was 13.2 months (SD four months). All patients except one responded to their questionnaire at one-year follow-up and X-rays were taken.

Radiographic union and malunion

Follow-up X-rays were done at one-year follow-up. There was 100% radiographic union in all patients, with no patient showing malunion or implant loosening.

Mobilization and length of stay

Patients were mobilised on different days subjecting to all factors. In subgroup analysis, patients in the younger age group were mobilized on day one, and in the older group (≥65 years) they were mobilized on the second day.

The mean length of hospital stay (LOS) for the entire cohort was 12.2 days (SD 5.6). Among patients aged ≥65 years, the mean LOS was 9.9 days (SD 6.09), while younger patients had a mean LOS of 8.33 days (SD 4.21).

Preoperatively, none of the patients required assistance from another individual for mobility. At one-year follow-up, 24 (75%) patients demonstrated no change in their use of mobility aids compared with pre-injury status. Two patients (6.2%) progressed from requiring no aids preoperatively to using a single-point walking stick, and one patient (3.1%) transitioned from using a four-wheeled walker pre-injury to requiring a wheelchair postoperatively. Overall, 29 (90.6%) of patients remained independently mobile at follow-up.

Morbidity and mortality

Postoperative complications were observed in six (18.75%) patients. Four patients (12.5%) required return to the operating theater due to postoperative infection. One patient (3.1%) developed implant-related irritation and subsequently underwent removal of the distal femoral plate at one year following the index procedure. Additionally, one patient (3.1%) experienced early postoperative medical complications, including pulmonary embolism diagnosed on postoperative day one, hospital-acquired pneumonia, and urosepsis. All complications occurred in patients aged ≥65 years.

Patient-reported outcomes

PROMs assessed using the EQ-5D-5L questionnaire at one-year follow-up are summarized in Table 3 for patients aged <65 years and in Table 4 for those aged ≥65 years. Overall, 21 (65.6%) of patients described no issues with mobility and a further seven (21.8%) reported only slight issues with mobility. Regarding self-care, 24 (75%) described no problems and six (18.7%) reported slight issues. The majority (29, 90.6%) of patients reported no or slight problems with usual daily activities. Eighteen patients (56.2%) reported no pain, with one patient (3.1%) describing severe pain. The overall EQ- 5D-5L mean index value was 0.713 (SD 0.200); the younger cohort scored 0.767 (SD 0.158) and the older cohort scored 0.691 (SD 0.222).

**Table 3:** Distribution of EuroQol five-dimension five-level (EQ-5D-5L) Responses Across Individual Domains in Patients Younger Than 65 Years EQ-5D-5L [14]

Domain	Mobility	Self-care	Usual activities	Pain/discomfort	Anxiety/depression
						1. No issues	6	5	4	5	7
2. Slight issues	1	3	4	3	0
3. Moderate issues	0	0	0	0	1
4. Severe issues	0	0	0	0	0
5. Extreme issues	1	0	0	0	0

**Table 4:** Distribution of EuroQol five-dimension five-level (EQ-5D-5L) Responses Across Individual Domains in Patients Older Than 65 Years EQ-5D-5L [14]

Domain	Mobility	Self-care	Usual activities	Pain/discomfort	Anxiety/depression
						1. No issues	15	19	5	13	16
2. Slight issues	6	3	17	6	6
3. Moderate issues	1	1	1	4	2
4. Severe issues	1	0	1	1	0
5. Extreme issues	1	1	0	0	0

## Discussion

Main findings

There is evidence in small clinical studies showing good results with naila and plate construct in distal femur fractures. However, there are only a few studies analysing malunion or PROMs in this construct.

Like other published data on NPCs, our study also reveals 100% union [15-17]. There were no malunion or implant backout observed in this study. Studies have shown malunion and nonunion rates in cases operated on with an isolated LP or IMN construct.

Nail fixation has the advantage of minimal fracture disruption during implant placement, and, with that, reaming stimulates healing, while plates are useful in osteoporotic patients having strong biochemical properties [18]. Studies on biochemical properties have shown that load-bearing devices are more prone to implant failure than IMN, while in the same study, it was found that there is a high risk of cortical failure in fixation done with load-sharing devices [17]. The malunion rate using isolated LP and IMN devices was found to be 7.6% and 16.4%, respectively. The nonunion rate in using isolated LP and IMN devices was found to be 8.8% and 3.6%, respectively [10].

Liporace et al. had described that energy can be properly distributed by combining a lateral locking construct with load sharing implant centrally [17]. Also, when both these implants are joined using locking screws, the femur can be spanned, and early weight bearing can be promoted due to the easy transition of forces. Kontakis et al. also explained the benefit of providing better rotational stability with NPC as compared to using these implants alone [19]. It was observed in biochemical studies that the risk of implant failure and malunion was higher in isolated implant failure than in NPC. It has also been shown in various studies that there has been 100% union rate in distal femur fractures fixed using NPC fixation in follow-up [15,17].

Patient-reported outcome measures

Mean value of EQ-5D-5L index was 0.731 (SD O.200) at one-year follow-up. It was found that in patients aged ≥65 years, the mean was 0.691, which was better as compared to the other study showing an index value < 0.4 in both IMN and LP used as a single implant at four months postoperative follow-up [13].

In our study, patients with age <65 years of age had a mean index value of 0.767 in one-year follow-up, which is comparable to the value of <0.76 found in a similar one-year study using a single IMN or LP construct [20]. Even though there are very few studies for comparison, the result indicates that PROMs in this NPC are superior to single construct fixation.

Mobility

There has been no change in mobility of the majority of patients from pre- to postoperative status. 65.6% patients have shown no problem in mobility in one-year follow-up, while one-quarter reported only slight issues. There was no issue noted in the self-care of the majority of patients, while most patients had noted no or slight problems with normal daily activity. Most of the patients had returned to their usual activity as related to their pre-morbid condition. This suggests that the NPC provides better post-operative rehabilitation for weight-bearing mobilisation.

The majority of patients regained independent mobility and were able to perform activities of daily living with little or no limitation. This outcome likely reflects the ability of NPC fixation to permit early weight bearing, thereby facilitating more effective rehabilitation and a return to pre-morbid functional status.

Complications

Although radiological union was achieved in all patients, postoperative complications occurred in 18.75% of the cohort, with 12.5% requiring return to the operating theater for deep infection. Notably, all complications occurred in patients aged ≥65 years. This infection rate is higher than that reported in several large series of isolated plating or intramedullary nailing, where deep infection rates typically range between 3-9%, depending on fracture complexity and patient comorbidity [10,15]. The relatively elevated infection rate in our series may reflect the advanced age, comorbidity burden, and fracture complexity within the elderly subgroup, as well as the small cohort size in which a few adverse events substantially influence percentage calculations. These findings highlight that while the NPC may provide a reliable union, the associated morbidity, particularly in elderly patients, must be carefully considered when selecting a fixation strategy.

Limitations

This study has several limitations. First, it was conducted at a single center with a relatively small sample size, limiting generalizability. Second, the absence of a control group treated with isolated lateral plating or intramedullary nailing precludes direct comparative analysis. Third, a formal a priori sample size calculation was not performed, as the study was exploratory in nature. Fourth, radiographic evaluation was not blinded, which may introduce assessment bias. Fifth, although the modified RUST scoring system was used to standardize union assessment, radiographic interpretation remains partially subjective despite predefined criteria. Finally, subgroup analyses were descriptive only and not powered for statistical comparison.

## Conclusions

NPC fixation for acute distal femur fractures demonstrated a 100% radiological union rate with no cases of malunion at one-year follow-up. Early postoperative mobilization was achievable in most patients, and PROMs indicated satisfactory functional recovery. However, postoperative complications occurred in 18.75% of patients, with 12.5% requiring reoperation for deep infection, and all adverse events were observed in the elderly subgroup (≥65 years). These findings suggest that while the NPC provides stable fixation and favorable union outcomes, the complication profile, particularly in older patients, must be carefully considered. Larger, comparative studies are required to further define the risk-benefit profile of this technique across different age groups.
